# Assessing intra- and inter-analyser imprecision in automated hematological laboratories

**DOI:** 10.5937/jomb0-62919

**Published:** 2026-02-27

**Authors:** Giuseppe Lippi, Marco Tosi, Mariateresa Rizza, Gian Luca Salvagno, Laura Pighi

**Affiliations:** 1 University of Verona, Section of Clinical Biochemistry, Verona, Italy

**Keywords:** laboratory haematology, automation, imprecision, laboratorijska hematologija, automatizacija, nepreciznost

## Abstract

**Background:**

Reliable haematology results are crucial for patient diagnosis and monitoring. Maintaining low variability is particularly important for key parameters such as haemoglobin, white blood cells, and platelets, especially in automated laboratory workflows where multiple haematological analysers are connected in the same line.

**Methods:**

Two residual whole blood samples, one normal and one pathological, were analysed in ten consecutive replicates on a Sysmex XN-10 analyser (XN-1) and then on a second connected analyser (XN-2). Intra-analyser and inter-analyser imprecision were calculated as coefficients of variation (CV%).

**Results:**

Intra-analyser CVs for the normal sample ranged from 0.3% for haemoglobin to 1.5% white blood cells, while inter-analyser CVs remained below 2%. For the pathological sample, intra-analyser CVs ranged from 0.3% to 1.0%, and inter-analyser CVs reached 2.1% for hematocrit and platelets. Red blood cell count, mean corpuscular volume, and neutrophils showed CVs &lt;1.9%. Higher variability was observed for low-abundance populations such as eosinophils and basophils (up to 20%).

**Conclusions:**

Sysmex XN-10 analysers exhibit strong intraand inter-analyser precision for most routine haematology tests, supporting their routine use in automated haematological lines.

## Abstract (sr)

**Uvod:**

Pouzdani hematoloski rezultati su od ključnog značaja za dijagnostiku i pracenje pacijenata. Održavanje niske varijabilnosti je posebno važno za ključne parametre kao sto su hemoglobin, leukociti i trombociti, naročito u automatizovanim laboratorijskim tokovima rada gde je vise hematoloskih analizatora povezano u istoj liniji.

**Metode:**

Dva rezidualna uzorka pune krvi, jedan normalan i jedan patoloski, su analizirana u deset uzastopnih ponav-ljanja na analizatoru Sysmex XN-10 (XN 1), a zatim na drugom povezanom analizatoru (XN 2). Intra-analizatorska i inter-analizatorska nepreciznost izračunate su kao koefici-jenti varijacije (CV%).

**Rezultati:**

Intra-analizatorski CV za normalan uzorak kretali su se od 0,3% za hemoglobin do 1,5% za leukocite, dok su inter-analizatorski CV ostali ispod 2%. Za patoloski uzorak, intra-analizatorski CV su se kretali od 0,3% do 1,0%, a inter-analizatorski CV dostigli su 2,1% za hematokrit i trom-bocite. Broj eritrocita, srednji zapreminski volumen eritro-cita i neutrofili pokazali su CV &lt;1,9% . Veca varijabilnost uočena je kod populacija niske zastupljenosti, kao sto su eozinofili i bazofili (do 20%).

**Zaključak:**

Analizatori Sysmex XN-10 pokazuju visoku intrai inter-analizatorsku preciznost za vecinu rutinskih hematoloskih testova, sto podržava njihovu rutinsku upotrebu u automatizovanim hematoloskim linijama.

## Introduction

The generation of accurate test results for haematology testing is essential for the diagnosis and monitoring of a wide range of haematological and non-haematological conditions [1]. Minimising assay imprecision is essential for generating reliable laboratory values that clinicians can confidently use to guide patient management. This is particularly critical for key parameters such as haemoglobin (Hb), white blood cell (WBC) and platelet counts, as well as for other routine measurements that serve as important diagnostic and prognostic indicators [2].

The importance of low imprecision in laboratory haematology has been magnified by the widespread adoption of integrated automation in modern clinical laboratories. Contemporary systems connect multiple analysers in sequential processing lines, enabling high-throughput testing, but introducing challenges in maintaining inter-analyser consistency [3]. Uniform low imprecision across all analysers is critical to ensure accurate longitudinal monitoring, irrespectiveof which analyser performs the measurement [4]. This study was therefore designed to evaluate the intra- and inter-analyser imprecision of routine haematological parameters measured by two sequential haematological analysers within the same interconnected automation line.

## Materials and methods

Two patient samples, collected in 3.0 mL K_2_EDTA blood tubes (Vacutest Kima, Padova, Italy),were randomly selected from all routine haematology specimens received by the Laboratory Medicine Service of the University Hospital of Verona for standard haematological testing during a normal working day. The first sample was from a healthy blood donor with no significant abnormalities in standard haematological parameters. The second sample was selected for exhibiting a high number of abnormalities on routine haematology tests. After completing routine analyses, both samples were measured in ten consecutive replicates on a first Sysmex XN-10 analyser (Sysmex Corporation, Kobe, Japan; hereafter referred to as »XN 1«), and subsequently reanalysed in ten consecutive replicates on a second Sysmex XN-10 analyser (hereafter referred to as »XN 2«), directly connected to the first analyser within a Sysmex proprietary automation line. Major details on the technical and analytical characteristics of Sysmex XN are available elsewhere [5].

Analytical imprecision was expressed as the coefficient of variation (CV%), calculated separately for each Sysmex XN analyser using the respective ten replicates (intra-analyser imprecision). Inter-analyser imprecision was instead computed from the combined set of the twenty consecutive replicates generated by both Sysmex XN analysers for each sample. The resulting imprecision was directly compared with the desirable total allowable error (TEa) reported in the Biological Variation Database of the European Federation of Clinical Chemistry and Laboratory Medicine (EFLM) (https://biologicalvariation.eu/) (Table 1). The whole blood samples used in this study were residual samples from routine testing and were fully anonymised prior to analysis; therefore, informed consent was not required. This study was conducted as part of a local validation of the local automated haematology line, using a protocol approved by the local Ethics Committee (approval number 971CESC; July 20, 2016).

**Table 1:** Intra- and inter-analyser imprecision of routine haematological testing on Sysmex XN analysers. CV%, coefficient of variation; SD, standard deviation; WBC, white blood cell count; RBC, red blood cell count; Hb, haemoglobin; Hct, hematocrit; MCV, mean corpuscular volume; MCH, mean corpuscular haemoglobin; RDW, red blood cell distribution width; MPV, mean platelet volume; Tea, total allowable error.

Parameter	TEa	Normal sample						Pathological sample					
		Intra-analyser<br>XN-1		Intra-analyser<br>XN-2		Inter-analyzer		Intra-analyser<br>XN-1		Intra-analyser<br>XN-2		Inter-analyzer	
		Mean<br>±SD	CV%	Mean<br>±SD	CV%	Mean<br>±SD	CV%	Mean<br>±SD	CV%	Mean<br>±SD	CV%	Mean<br>±SD	CV%
WBC<br>(x 10^9^/L)	14.2%	6.80±0.10	1.5%	6.61±<br>0.07	1.1%	6.70±<br>0.13	1.9%	13.57±<br>0.12	0.9%	13.30±<br>0.10	0.8%	13.44±<br>0.17	1.3%
RBC<br>(x 10^12^/L)	4.2%	6.03±<br>0.04	0.7%	6.07±<br>0.05	0.8%	6.05±<br>0.05	0.8%	3.15±<br>0.03	1.0%	3.12±<br>0.01	0.4%	3.13±<br>0.03	0.9%
Platelets<br>(x 10^9^/L)	11.0%	329±<br>5	1.4%	337±<br>5	1.6%	333±<br>6	1.9%	173±<br>2	1.0%	176±<br>5	1.6%	174±<br>4	2.1%
Hb (g/L)	3.3%	130.8±<br>0.6	0.5%	131.7±<br>0.6	0.5%	131.3±<br>0.8	0.6%	96.1±<br>0.3	0.3%	96.2±<br>0.6	0.6%	96.2±<br>0.5	0.5%
Hct	3.9%	0.416±<br>0.002	0.5%	0.403±<br>0.03	0.8%	0.410±<br>0.007	1.8%	0.307±<br>0.03	0.9%	0.295±<br>0.002	0.6%	0.301±<br>0.06	2.1%
MCV (fL)	1.7%	97.3±<br>0.5	0.5%	94.3±<br>0.4	0.4%	95.8±<br>1.5	1.6%	77.3±<br>0.5	0.7%	74.6±<br>0.5	0.7%	76.0±<br>1.5	1.9%
MCH (pg)	1.3%	27.5±<br>0.3	1.0%	27.8±<br>0.2	0.8%	27.6±<br>0.3	1.1%	30.6±<br>0.2	0.7%	30.8±<br>0.1	0.4%	30.7±<br>0.2	0.7%
RDW (CV%)	2.6%	12.62±<br>0.04	0.3%	12.57±<br>0.05	0.4%	12.60±<br>0.05	0.4%	15.13±<br>0.10	0.7%	15.19±<br>0.10	0.3%	15.16±<br>0.10	0.7%
MPV (fL)	3.8%	12.32±<br>0.12	0.9%	12.36±<br>0.07	0.5%	12.34±<br>0.10	0.8%	13.30±<br>0.11	0.8%	13.17±<br>0.08	0.6%	13.24±<br>0.12	0.9%
Neutrophils<br>(x 10^9^/L)	17.5%	4.90±<br>0.07	1.4%	4.77±<br>0.06	1.2%	4.84±<br>0.09	1.9%	6.91±<br>0.07	1.0%	6.80±<br>0.10	1.4%	6.86±<br>0.10	1.4%
Lymphocytes<br>(x 10^9^/L)	14.9%	1.44±<br>0.06	4.3%	1.40±<br>0.05	3.7%	1.42±<br>0.06	4.3%	2.08±<br>0.02	1.1%	2.05±<br>0.05	2.4%	2.06±<br>0.04	2.1%
Monocytes<br>(x 10^9^/L)	18.3%	0.39±<br>0.02	4.8%	0.38±<br>0.02	6.1%	0.38±<br>0.02	5.7%	0.53±<br>0.03	5.9%	0.52±<br>0.02	4.1%	0.52±<br>0.03	5.1%
Eosinophils<br>(x 10^9^/L)	29.7%	0.04±<br>0.01	17.1%	0.04±<br>0.01	21.3%	0.04±<br>0.01	19.4%	0.06±<br>0.01	18.9%	0.06±<br>0.01	23.3%	0.06±<br>0.01	19.0%
Basophils<br>(x 10^9^/L)	18.2%	0.02±<br>0.00	19.9%	0.02±<br>0.00	14.3%	0.02±<br>0.00	18.2%	0.04±<br>0.01	15.8%	0.04±<br>0.01	17.3%	0.04±<br>0.01	17.0%

## Results

**Figure 1:**
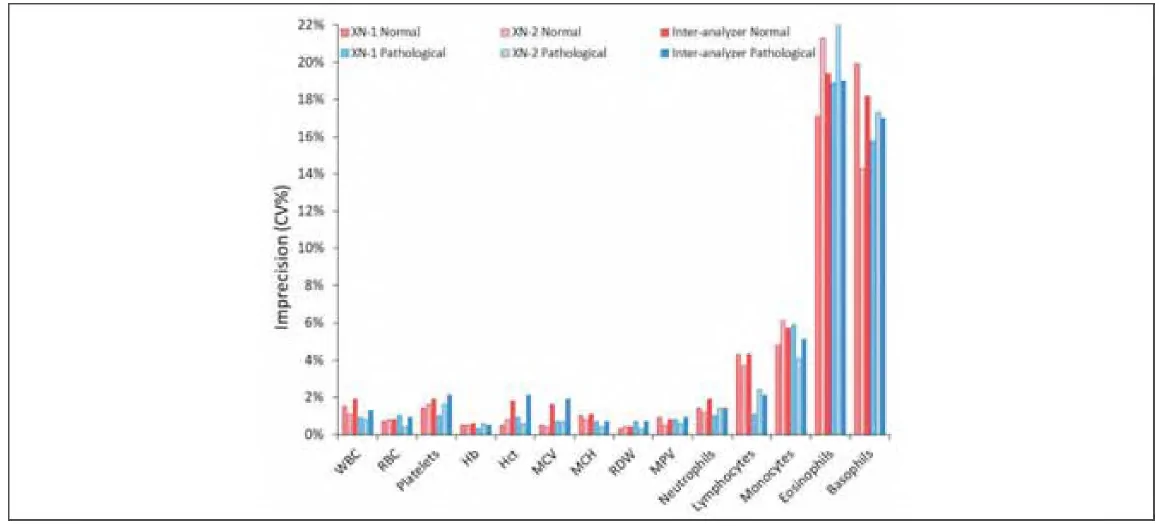
Intra- and inter-analyser imprecision of routine haematological testing on Sysmex XN analysers.<br>WBC, white blood cell count; RBC, red blood cell count; Hb, haemoglobin; Hct, hematocrit; MCV, mean corpuscular volume; MCH, mean corpuscular haemoglobin; RDW, red blood cell distribution width; MPV, mean platelet volume.

The results of this investigation are summarised in Table 1 and Figure 1. Intra-analyser imprecision (CV%) for routine haematological parameters measured by the two Sysmex XN-10 analysers (XN 1 and XN 2) demonstrated excellent to modest variability for normal and pathological blood samples. For the normal sample, intra-analyser CVs ranged from 0.3% for haemoglobin (Hb) to 1.5% for white blood cells (WBC), while inter-analyser imprecision remained similarly low, with CVs generally below 2%. In the pathological sample, intra-analyser CVs ranged from 0.3% for Hb to 1.0% for WBC, and inter-analyser CVs reached 2.1% for hematocrit (Hct) and platelets. Parameters such as RBC count, mean corpuscular volume (MCV), and neutrophil count also exhibited intra- and inter-analyser CVs typically below 1.9%. Intermediate imprecision was observed for lymphocytes and monocytes, whereas cell populations with low absolute counts, such as eosinophils and basophils, displayed much higher imprecision (up to around 20%), reflecting the predictable biological and analytical variability at low absolute counts. An imprecision greater than the current TEa threshold, as defined in the EFLM Biological Variation Database, was observed only for the MCV in inter-analyser assessment with the pathological sample (one instrument) and for the basophil count in intra-analyser assessment with the normal sample (one instrument).

## Discussion

The findings of this study indicate that the Sysmex XN-10 analysers exhibit robust intra- and inter-analyser precision across a broad spectrum of the most clinically used haematological parameters, supporting their integration into automated laboratory lines. Low coefficients of variation for critical clinical parameters such as WBC, platelet count and Hb underscore their reliability for routine diagnostics and longitudinal patient monitoring. The comparable precision between analysers confirms that the sequential processing of patient samples on interconnected devices does not appear to jeopardise the consistency and repeatability of test results. Except for MCV andbasophil counts, intra- and inter-analyser imprecision for all other haematological parameters fell within the current TEa thresholds defined by the EFLM Biological Variation Database. These findings suggest good overall analytical performance, with only two parameters exceeding acceptable limits.

Nevertheless, we found greater imprecision when assaying underrepresented cell populations,such as eosinophils and basophils, a finding aligned with the expected analytical challenges at low counts, already emphasised in several previous studies using these same analytical systems [6][7][8]. For example, Pérez et al. used the same analyser (i.e., Sysmex XN-series) and reported relatively low intra- and interassay imprecision for most parameters, with CVs below the EFLM TEa thresholds for WBC, red blood cells (RBC), platelets, Hb, Hct, MCV, red cell distribution width (RDW), and mean platelet volume (MPV). However, values exceeding the EFLM TEa were occasionally observed for mean corpuscular haemoglobin (MCH), lymphocytes, monocytes, eosinophils, and basophils [6]. Birindelli et al. also employed three Sysmex XN systems and observed inter-module imprecision below the EFLM TEa thresholds for WBC, RBC, Hb, Hct, MCV, and platelets, while the specific threshold was exceeded for the MCH parameter [7]. Given the high variability observed in rare cell populations, significant caution is hence warranted when performing longitudinal analyses of these parameters. This is particularly important, even if the primary source of imprecision is attributed to intra-analyser rather than inter-analyser variability, as subtle fluctuations may still affect clinical interpretation and decision-making over time.

In summary, Sysmex XN 10 analysers exhibit high precision across most routine haematological parameters, supporting their reliable use in clinical practice and integration into automated laboratory workflows. Additional studies should be planned to include a larger sample size, investigate sample stability over time, and the impact of variations in operating conditions (e.g., temperature, humidity, or instrument calibration).

## Dodatak

### Conflict of interest statement

All the authors declare that they have no conflict of interest in this work.

## References

[1] Hoffmann J J M L, Urrechaga E (2023). Recent advances in laboratory hematology reflected by a decade of CCLM publications. Clin Chem Lab Med.

[2] Vis J Y, Huisman A (2016). Verification and quality control of routine hematology analyzers. Int J Lab Hematol.

[3] Chhabra G (2018). Automated hematology analyzers: Recent trends and applications. J Lab Physicians.

[4] Vidali M, Carobene A, Apassiti E S, Napolitano G, Caracciolo A, Seghezzi M, Previtali G, Lippi G, Buoro S (2021). Standardization and harmonization in hematology: Instrument alignment, quality control materials, and commutability issue. Int J Lab Hematol.

[5] Seo J Y, Lee S T, Kim S H (2015). Performance evaluation of the new hematology analyzer Sysmex XN-series. Int J Lab Hematol.

[6] Pérez I, Redin M E, Vives A, Garrido A, Urrechaga E, Lacasta M (2016). Local verification between the hematological analyzers Sysmex XN-series and XE-5000. Int J Lab Hematol.

[7] Birindelli S, Aloisio E, Carnevale A, Brando B, Dolci A, Panteghini M (2017). Evaluation of long-term imprecision of automated complete blood cell count on the Sysmex XN-9000 system. Clin Chem Lab Med.

[8] Coussee A, Robbrecht J, Maelegheer K, Vandewal W, Florin L (2025). Evaluating the Performance of the New Sysmex XR-Series Haematology Analyser: A Comparative Study with the Sysmex XN-Series. LabMed.

